# Prognostic factors of Bell's palsy and Ramsay Hunt syndrome

**DOI:** 10.1097/MD.0000000000005898

**Published:** 2017-01-13

**Authors:** Zhengyi Cai, Huijing Li, Xun Wang, Xiaoting Niu, Peiqi Ni, Wanli Zhang, Bei Shao

**Affiliations:** a Department of Neurology; b Department of Pediatrics, First Affiliated Hospital of Wenzhou Medical University, Wenzhou, Zhejiang Province, China.

**Keywords:** Bell's palsy, electroneurography, prognostic factors, Ramsay Hunt syndrome

## Abstract

The aim of this study was to compare clinical characteristics, electroneurography (ENoG) results, and functional outcomes of patients with Bell's palsy (BP) and Ramsay Hunt syndrome (RHS).

Around 57 patients with BP and 23 patients with RHS were enrolled in this study from January 2010 and September 2015. Both clinical characteristics and ENoG results were recorded at hospital admission. The evaluations of functional outcomes were conducted with House–Brackmann (H-B) grading system at 6-month follow-up.

There were no significant differences in age, gender proportion, initial H-B grades, time before commencement of treatment and the presence of comorbid disease in 2 groups. However, the final H-B grades at 6-month follow-up were significantly better in BP patients than RHS patients. The results of ENoG showed that degeneration index (DI) was significantly higher in the RHS group than the BP group. But no significant difference was found in the value of prolonged latency time (PLT) between the 2 groups. In multivariate analysis, age and ENoG DI were independently associated with functional outcome of recovery in the BP group (OR 0.167, 95% CI 0.038–0.622, *P* = 0.009 and OR 0.289 95% CI 0.107–0.998, *P* = 0.050, respectively). However, in the RHS group, only ENoG DI was related to the final H-B grades (OR 0.067, 95% CI 0.005–0.882, *P* = 0.040). Spearman's rank correlation analysis showed that higher age and ENoG DI were related to poorer prognosis in 2 groups (*P* < 0.05). PLT was related to functional outcomes only in the BP group (*r*_*s*_ = 0.460, *P* < 0.001). The receiver operating characteristic (ROC) of ENoG DI analysis revealed that the cutoff value was 67.0% for BP prognosis and 64.5% for RHS prognosis. What's more, patients with hypertension or diabetes mellitus had both higher final H-B grade and ENoG DI than those without the same comorbidity.

Patients with RHS had poorer prognosis than those with BP. Some factors including age, ENoG DI, and the presence of disease influenced recovery from BP and RHS. The present study demonstrated that BP patients with ENoG DI < 67.0% and RHS patients with ENoG DI < 65.5% had a greater opportunity for recovery within half a year.

## 1 Introduction

Acute facial palsy (AFP) is mainly characterized by an acute peripheral and unilateral facial weakness (complete or incomplete). The estimated incidence of AFP is about 30 patients per 100,000 populations annually.^[1]^ Early diagnosis and accurate treatment for patients with AFP may accelerate recovery and prevent possible complications. Also, the questions most probably asked by these patients are whether their facial function will return to normal one day and how long this is going to take.

Although predictability of AFP outcomes is of essential importance for patient counseling and clinical management, the prognosis differs according to various etiologies. AFP can be caused by a variety of diseases, including Bell's palsy, Ramsay Hunt syndrome, trauma, cancer, and iatrogenic injury.^[2,3]^ Among these disease, Bell's palsy (BP) and Ramsay Hunt syndrome (RHS) are the most common causes.^[1–4]^ In the past, a large number of studies have been focused on prognostic factors of BP.^[5–7]^ Besides clinical predictors, electroneurography (ENoG) has also been implemented to determine prognosis.^[1,8–10]^

However, the predictive value of ENoG in RHS prognosis was less frequently reported.^[11]^ Therefore, in the present study, we compared clinical characteristics, ENoG results, and functional outcomes of patients with BP and RHS.

## 2 Materials and methods

The study population consisted of 57 patients with Bell's palsy and 23 patients with the Ramsay Hunt syndrome between January 2010 and September 2015. The study was approved by the Ethics Committee of First Affiliated Hospital of Wenzhou Medical University. Informed consent was obtained from all the subjects.

Patients were diagnosed with BP if no other causes of facial palsy could be identified. Patients were diagnosed with RHS if they had peripheral facial palsy or lesions on the external auditory canal or the vesicles around the ear. Recorded clinical data included gender, age, time from first onset to initial treatment, and the presence of common comorbid disease such as hypertension (HTN) or diabetes mellitus (DM). The House–Brackmann (H-B) grading system^[12]^ was used to assess initial facial nerve impairment (the worst grade evident after facial palsy had developed) and final facial nerve impairment (measured 6 months at follow-up). All patients underwent ENoG at hospital admission. The ENoG Degeneration Index (DI) was calculated as: [100 – (ENoG amplitude affected/unaffected side) × 100]. The prolonged latency time (PLT) was calculated as: [latency time (affected – unaffected side)].

Patients were treated with a standard therapy protocol once they meet the inclusion criteria. The medical treatment included oral prednisone (30 mg/d for 5 days, followed by 5 days of tapering), oral acyclovir (0.2 g/tid for 7–10 days accordingly), and neurotrophic drugs (mecobalamin tablet 0.5 mg/tid, and fursultimine tablet 50 mg/tid for 30–60 days accordingly). Eye care was implemented when there was inadequate eye closure, which included artificial tears during the day and an ointment at night. Patients were also educated to keep the eyelid closed with tape or use a moisture chamber while sleeping. For those who preferred physical therapy, acupuncture was also recommended.

SPSS 19.0 software (Chicago, IL) was used for statistical analysis. Continuous variables were expressed as mean ± standard deviation (SD) or median (interquartile range, IQR) depending on their normal distribution. The results were indicated as percentages for categorical variables. The normally distributed variables were compared using Student's *t* test and the asymmetrically distributed variables were compared using the Mann–Whitney *U* test. The Chi-square test was used to compare proportions. The relation between ENoG DI and functional outcome of facial nerve after 6 months was computed by multivariate logistic regression analysis. Results were expressed as adjusted odds ratios (ORs) (95% confidence intervals, CIs). Spearman's rank correlation was performed for bivariate correlation and a receiver operating characteristic (ROC) was conducted to assess the accuracy of ENoG as a predictor for 2 disorders. The *P* value less than 0.05 was deemed statistically significant.

## 3 Results

Demographic and clinical features of BP and RHS patients are detailed in Table 1.

**Table 1:**
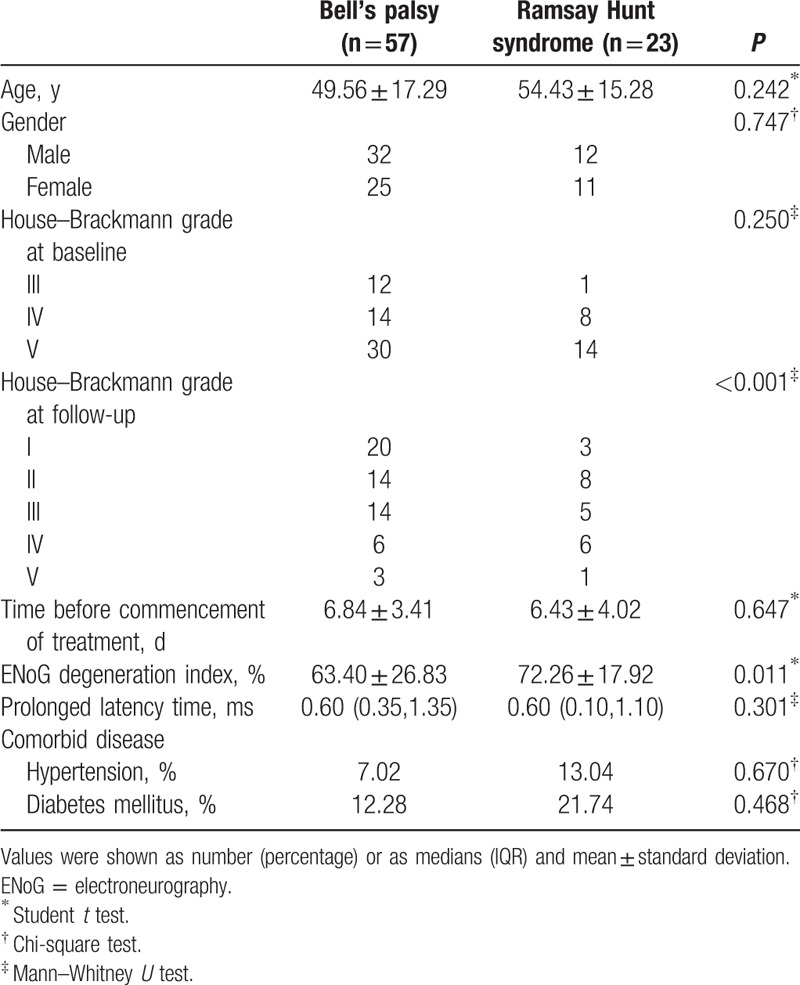
Demographic, clinical, and neurophysiological characteristics of patients.

There were no significant differences in age, gender proportion, initial H-B grades, time before commencement of treatment, and the presence of comorbid disease in 2 groups (*P* > 0.05). However, the final H-B grades at 6-month follow-up were significantly better in BP patients than RHS patients (*P* < 0.001). The results of ENoG showed that DI was significantly higher in the RHS group than the BP group (*P* = 0.011). But no significant difference was found in the value of PLT between 2 groups (*P* = 0.301).

**Figure 1:**
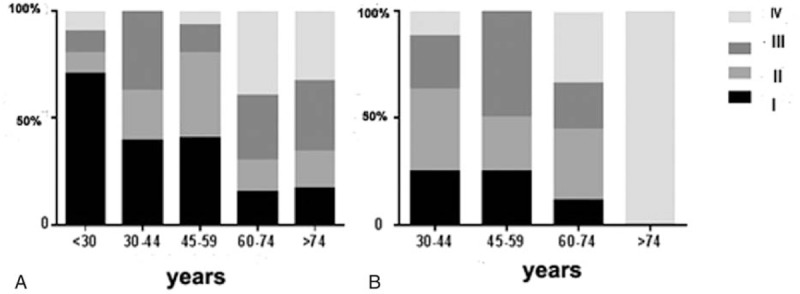
Classification of facial nerve recovery according to age in (A) the BP group and (B) the RHS group. BP = Bell's palsy, RHS = Ramsay Hunt syndrome.

A multivariate logistic model was set up and 5 clinical or neurophysiological determinants of final H-B grades for acute facial palsy were included (e.g., age, gender, time before commencement of treatment, Initial H-B grades, ENoG DI). Logistic analysis revealed that in BP group, age, and ENoG DI were independently associated with functional outcome of recovery (OR 0.167, 95% CI 0.038–0.622, *P* = 0.009 and OR 0.289 95% CI 0.107–0.998, *P* = 0.050, respectively). However, in the RHS group, we found that only ENoG DI was related to final H-B grades (OR 0.067, 95% CI 0.005–0.882, *P* = 0.040). To better analyze the prognosis according to the patients age, we divided patients into 5 age groups: < 30 years, between 30 and 44 years, between 45 and 59 years, between 60 and 74 years, ≥ 75 years. Spearman's rank correlation analysis showed that higher age was related to poorer prognosis in 2 groups (*P* < 0.05, results were not shown). It was also obvious that prognosis were better in the BP group than the RHS group at different age stages (Fig. 1).

**Figure 2:**
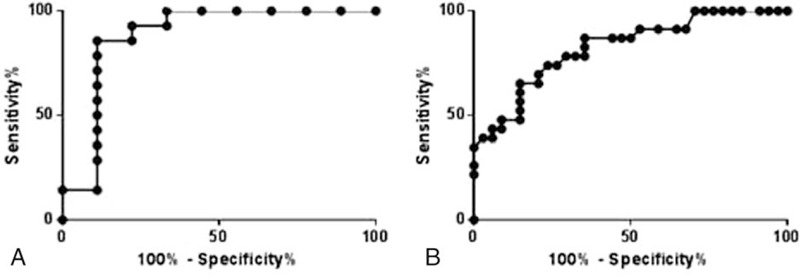
ROC curves to evaluate the utility of ENoG DI for prediction of recovery in (A) the BP group and (B) the RHS group. BP = Bell's palsy, DI = degeneration index, ENoG = electroneurography, ROC = receiver operating characteristic, RHS = Ramsay Hunt syndrome.

According to the results of ENoG, ENoG DI was subdivided into 3 levels (< 70%, 70–90%, > 90%) and PLT was divided as 5 subgroups; ≤0.5 ms, between 0.5 and 1.0 ms, between 1.0 and 1.5 ms, between 1.5 and 2 ms, > 2 ms. Consequently, ENoG DI was remarkably consistent with prognosis in both BP and HRS groups (*r*_*s*_ = 0.489, *P* < 0.001 and *r*_*s*_ = 0.691, *P* < 0.001; Table 2). However, PLT was related to functional outcomes only in the BP group (*r*_*s*_ = 0.460, *P* < 0.001; Table 3). The ROC of ENoG DI analysis revealed that the cutoff value was 67.0% for BP prognosis and 64.5% for RHS prognosis (Fig. 2).

**Table 2:**
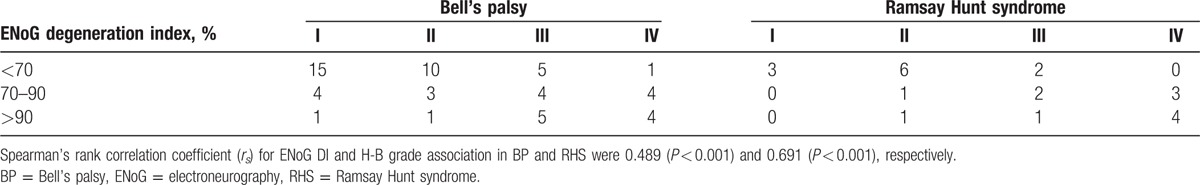
Association between results of ENoG and House–Brackmann grades at follow-up in patients with Bell's palsy and Ramsay Hunt syndrome.

**Table 3:**
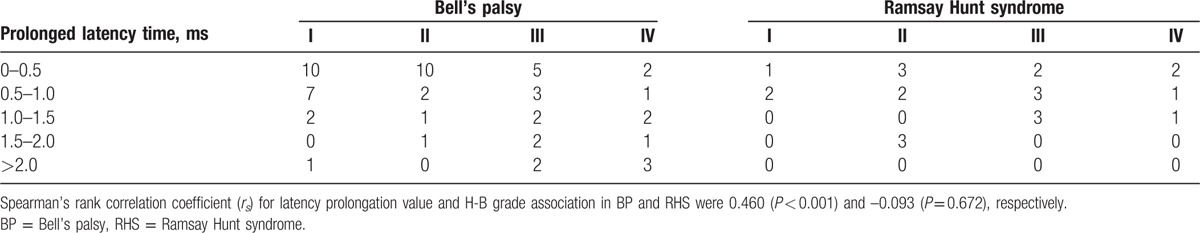
Association between value of prolonged latency time and House–Brackmann grades at follow-up in patients with Bell's palsy and Ramsay Hunt syndrome.

Furthermore, we also analyzed the influences of HTN or DM on the prognosis of the 2 disorders (Table 4). In BP, there was no significant difference in H-B grade at baseline between the HTN group and non-HTN group (*P* = 0.500), as well as between the DM group and non-DM group (*P* = 0.482). However, the H-B grade at follow-up was significantly better in the non-HTN group than the HTN group (*P* = 0.013), and in the non-DM group than DM group (*P* = 0.012). ENoG DI was higher in the HTN group than the non-HTN groups (*P* = 0.009), and in the DM group than the non-DM group (*P* = 0.008).

**Table 4:**
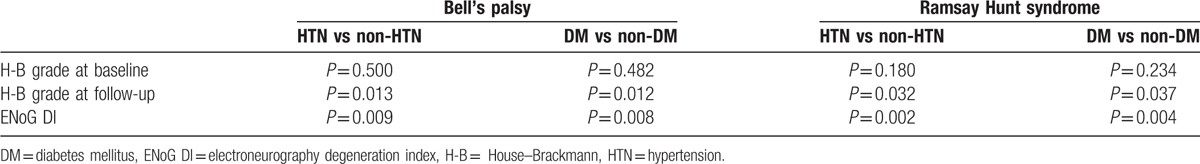
Influence of comorbidities on prognosis of patients with Bell's palsy and Ramsay Hunt syndrome.

Similarly, in RHS, there was also no significant difference in the H-B grade at baseline between the HTN group and non-HTN groups (*P* = 0.180), as well as between the DM group and the non-DM group (*P* = 0.234). However, the H-B grade at follow-up was significantly better in the non-HTN group than the HTN group (*P* = 0.032), and in the non-DM group than the DM group (*P* = 0.037). ENoG DI was higher in the HTN group than the non-HTN groups (*P* = 0.002), and in the DM group than the non-DM group (*P* = 0.004).

## 4 Discussion

To date, it is acknowledged that ENoG can be used to objectively measure the degree of nerve injury and predict the prognosis of AFP.^[1]^ The ENoG records compound muscle action potential (CMAP) of facial muscle, via stimulating transcutaneously at the stylomastoid foramen using a bipolar stimulating. On the one hand, the ENoG DI can reflect the percentage of facial nerve injury of the paralyzed side, which is caused by axonal degeneration or conduction block. On the other hand, the PLT can show prolongation in conduction time of the paralyzed nerve, which is caused by acute inflammatory or demyelination. On the whole, the combined use of DI and PLT may evaluate the degree, type, and recovery chance of facial neural injury. Similar with a previous study,^[13]^ our results showed that ENoG DI was significantly higher in patients with RHS than BP in the acute phase, indicating that damage to infected nerves was more severe in RHS than BP. It also suggested that ENoG may be a more sensitive tool to give information on the paralysis status, especially with regard to the treatment and prognosis.

By comparison of final H-B grades between 2 groups, our study demonstrated that prognosis was better in BP than RHS, which was in agreement with previous studies.^[14,15]^ What's more, logistic regression analysis in our study revealed that ENoG DI was an important prognostic factor for both BP and RHS. This neurophysiological parameter was also highly consistent with H-B grades. Byun et al^[16]^ carried out a study of 66 BP patients and 22 RHS patients and confirmed the significant effect of ENoG value on recovery in 2 disorders. However, Ozgur et al^[8]^ collected ENoG data within 7 days from onset of symptoms but found no significant correlation between patients with ENoG ≥ 75% and the patients with H-B III or higher at the 3-month staging. Lee et al^[11]^ investigated the ENoG value between 7 and 10 days for BP, and between 10 and 14 days for RHS, and found that it was not accurate or reliable enough to determine the prognosis of facial paralysis quantitively. Fisch^[17]^ showed that the degree of nerve degeneration was predictive of recovery as determined by ENoG. Generally, patients with greater than 90% loss of amplitude within the first 10 days had poorer recovery. A multicenter study showed that surgical decompression could prevent an adverse outcome in patients with poor prognostic findings on ENoG.^[18]^ Thus, it is once recommended that ENoG DI > 90% be an established electrophysiologic criteria for surgery. However, Mantsopoulos et al^[19]^ found that 9.3% of patients with <90% DI still showed an incomplete recovery. What's more, the ENoG cutoff value in our study is also different from others.^[11,20]^ From this regard, for the patients with ENoG DI between 65% and 90%, it is doubtful that whether a surgical treatment is necessary. All in all, further research is required to confirm conclusions and hypothesis mentioned above, since there are biases in patient selections, the examination time from the onset of symptoms, definition of functional recovery, and follow-up time.

There was significant correlation between the value of PLT and poor outcome of BP but not RHS. Also in our study, there was no significant difference in initial PLT values between BP and RHS. We speculated that it may result from different underlying pathogenesis of 2 disorders. RHS is caused by latent varicella-zostervirus infection reactivated in the geniculate ganglion, which will cause otalgia, pinnal vesicular rash, and peripheral facial palsy.^[21]^ The lesions include the eardrum, the external auditory canal and the central portion of the ear, the cavum conchae and individual fibers of the facial nerve.^[22]^ On the one hand, the nerve injury is much more severe in the acute phase of RHS, so the recovery of facial nerve function is less satisfactory. On the other hand, PLT represents demyelination degree of facial nerve outside the stylomastoid foramen. If demyelination is not as severe as axon damage, latency time of paralyzed side can remain normal with obviously changed ENoG DI. Thus, during analysis of electrophysiological measure in predicting prognosis of AFP, latency time is not of primary importance, especially for RHS patients.

In addition, we took other prognostic factors such as age and coexisting diseases into consideration for statistical analysis. The prognosis of patients with BP and RHS may both be related to patient age at onset. To be specific, with increasing age, prognosis of BP or RHS became poorer respectively. And no matter in which age group, the final H-B grades were higher in RHS patients than BP patients. Danielidis et al^[23]^ attributed this phenomenon to a decrease of peripheral blood supply in older patients due to an increase in vascular degeneration. However, Yeo et al^[15]^ found that only in the RHS group, older patients had a more severe final status and lower chance of making a complete recovery. In other words, the age was a prognostic factor for RHS but not BP in his study. When we assessed the H-B grade and ENoG DI with respect to the presence of a comorbid disease, we found that BP/RHS patients with HTN or DM had both higher final H-B grade and ENoG DI than those without the same comobidity. Our results imply that HTN and DM may affect neural functional recovery. In contrast, Yeo et al^[15]^ demonstrated that HTN and DM are factors influencing the prognosis of RHS but not BP. Experimental animal models suggested that the diabetic group had significantly increased incidence of facial nerve paralysis and more severe nerve damage when compared with the nondiabetic group.^[24]^ We obtained similar results that functional outcomes were worse in patients with comorbid diseases than those without comorbid diseases. In particular, patients with BP or RHS and comorbid DM had poorer prognosis than did those with HTN, which may be attributable to diabetic neuropathy^[3]^ or herpes-simplex virus infection amplified by diabetes mellitus.^[24]^ Although *in vitro* studies mentioned above may explain our results, the conflicting results between clinical observations in different centers need further confirmation.

There are several limitations in our study. First, the number of cases was limited, especially the number of RHS patients was relatively small. Second, because all patients underwent ENoG at admission, there was bias in the electrophysiological examination time from symptom onset. Third, the study subjects came from a single center. Thus, our results may not be generalized to patients in other areas.

In conclusion, patients with RHS had poorer prognosis than those with BP. Some factors including age, ENoG DI, and the presence of disease influenced recovery from RHS and BP. The present study demonstrated that BP patients with ENoG DI < 67.0% and RHS patients with ENoG DI < 65.5% had a greater opportunity for recovery within half a year.

## Acknowledgments

The authors thank Jie Pan for expert technical assistance in carrying out electroneurography tests.
